# Efficacy of modified versus standard Valsalva maneuvers on clinical outcomes and satisfaction of children with paroxysmal supraventricular tachycardia: randomized control trial

**DOI:** 10.1186/s12887-025-06396-9

**Published:** 2025-12-17

**Authors:** Nagwa Ramadan Esmail Magor, Seham Eid Hashem Elhalafawy, Samar Eldesoky Mohamed Ads, Mohamed Elsayed Abdelfattah, Sahar Wasfy Mahmoud Melika

**Affiliations:** 1 Present Address: Pediatric Nursing Department, Faculty of Nursing, Tanta University, Tanta, Egypt; 2 Cardiology Department, Faculty of Medicine, Kafr Elsheikh University, Kafrelsheikh, Egypt; 3 Pediatric Nursing Department, Faculty of Nursing, Kafr Elsheikh University, Kafrelsheikh, Egypt

**Keywords:** Children clinical outcomes, Paroxysmal supraventricular tachycardia, Modified/standard valsalva maneuvers, Satisfaction, Randomized control trial

## Abstract

**Introduction:**

Valsalva maneuvers are the initial line in management of paroxysmal supraventricular tachycardia in hemodynamically stable children. This study aimed to compare the efficacy of modified versus standard Valsalva maneuvers on the clinical outcomes and satisfaction of children with paroxysmal supraventricular tachycardia.

**Methods:**

The study used randomized controlled trial and recruited ninety children with paroxysmal supraventricular tachycardia from Pediatric Emergency Department and Pediatric Cardiac Intensive Care Unit at Tanta University Hospitals, El-Gharbia Governorate, Egypt. The researchers divided the studied children into three equal groups of thirty. A control group that received conventional hospital care, an intervention group I that received modified Valsalva maneuver plus conventional hospital care, and an intervention group II that received standard Valsalva maneuver plus conventional hospital care. The primary outcome was the return to sinus rhythm within the first 5 min of admission and the secondary outcomes were decreased dyspnea, decreased antiarrhythmic therapy use, length of stay time in hospital as well as children’s satisfaction.

**Results:**

More than half (53.3%) of the children who received the modified Valsalva maneuver returned to sinus rhythm within the first five minutes post-implementation compared to 33.3% of the children who received the standard Valsalva maneuver. Children within modified Valsalva maneuver group had a mean satisfaction score of 25.56 ± 1.67 that was significantly higher than those in the standard Valsalva maneuver group’s score of 20.10 ± 2.57 (*P* = 0.0001).

**Conclusion:**

The modified version of the Valsalva maneuver was significantly more effective than the standard Valsalva maneuver in terminating supraventricular tachycardia and improving children’s clinical outcomes. This included a decrease in the degree of dyspnea within the first minute from severe to moderate and reducing the need for administering antiarrhythmic drugs for management of SVT episodes. Additionally, children in the MVM group had a higher mean satisfaction score than those in the SVM group, with highly statistically significant differences.

**Trial registration:**

PACTR202407479098909. Registered 15/07/2024.

**Supplementary Information:**

The online version contains supplementary material available at 10.1186/s12887-025-06396-9.

## Introduction

Paroxysmal supraventricular tachycardia (PSVT) is the most common form of arrhythmia in children, estimated to occur in 1 in 250 to 1 in 1000 children, with peaks, one within the first year and the others approximately 5 to 7 years of age as well as during adolescence [1]. Paroxysmal supraventricular tachycardia refers to an abnormally rapid heart rhythm above the ventricles, characterized by a narrow QRS complex with a sudden onset and end. While, it usually occurs at rest, there are some triggers that can lead to episodes of SVT which includes medications such as asthma relievers and flu medicines, excessive caffeine, and stress or emotional upset [2, 3].

**Fig. 1:**
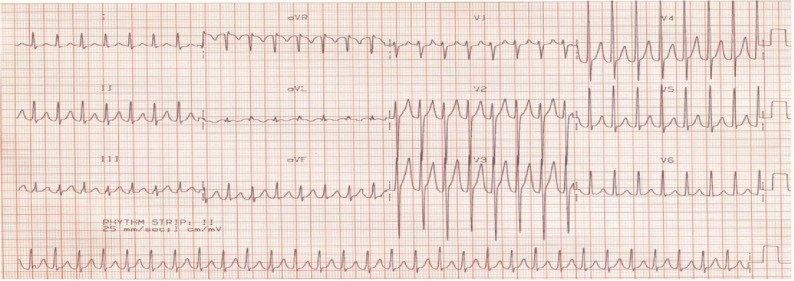
a 12 leads ECG shows regular narrow complex tachycardia at rate about 180 bpm (during SVT attack) for a real case in this study

In pediatric patients, the mechanisms of SVT include atrioventricular re-entry tachycardia, which accounts for more than 70% of cases, followed by ectopic atrial tachycardia, atrioventricular nodal re-entry tachycardia (AVNRT), and abnormal automaticity [4, 5]. The most common symptoms in children include palpitations, chest pain, dizziness, pallor, sweating, and dyspnea. Adolescents commonly display all of these symptoms along with perspiration, fatigue, and anxiety [6]. The diagnostic tests include electrocardiogram (ECG), Holter monitors, exercise stress testing, and electro-physiologic studies. A 12-lead (ECG) recorded during an ongoing attack commonly shows abnormal undetectable QRS complex and P wave as well as regular rhythms with tachycardia of a rate >180 bpm in older children and adolescents (Fig. 1) and >220 bpm in infants [7].

Supraventricular tachycardia can lead to serious complications, such as tachycardia induced cardiomyopathy. However, some children with cardiac comorbidities may lead to exacerbated congestive heart failure, angina, or even sudden death [3]. Management strategies for stable PSVT include nonpharmacological approaches such as vagal maneuvers alongside pharmacological antiarrhythmic drugs such as adenosine and calcium channel blockers. Current European guidelines recommend applying of vagal maneuvers before administering antiarrhythmic drugs in hemodynamic stable children [2, 8].

Vagal maneuvers typically act by raising parasympathetic tone, which prolongs the refractory period of the atrioventricular node and terminates the episodes of SVT. Pediatric patients typically respond better to vagal maneuvers, with 30%−60% of children responding compared to 17% of adults. Also, modified Valsalva maneuver was succeeded in restoring the sinus rhythm, in 50% of children with PSVT in the first attempt as mentioned by Mesía et al. (2023) [9, 10]. The Valsalva maneuver is a commonly used vagal maneuver, which is considered one of the simplest, easiest, and most effective techniques for restoring sinus rhythm [11, 12]. The Standard Valsalva Maneuver (SVM) involves a sustained expiration against a closed glottis for 15–20 s, with the patient remaining in a semi-recumbent position during and immediately after [13]. The Modified Valsalva Maneuver (MVM) involves a change in the body posture immediately after releasing the strain, the patient assumes a flat body position accompanied with a 15-second passive leg lift to improve venous return and vagal activation during the relaxation phase [14].

Caring of children with PSVT presents a challenge for the emergency pediatric nurses and requires advanced clinical skills to accurately assess the hemodynamic status. Nurses are also responsible for obtaining detailed history, monitoring, and interpreting the (ECG) accurately before, during, and after treatment, and administering the prescribed antiarrhythmic medications. In addition, nurses can perform interventions such as assisting in the application of Valsalva maneuvers for children experiencing PSVT [8, 15, 16]. Satisfaction is a multifaceted concept and considered as a gold standard of nursing care. Nurses are also responsible for delivering high-quality nursing care that could achieve the satisfaction of the children [17].

Although evidence supports the effectiveness of the Valsalva maneuvers in terminating SVT in children, studies evaluating their effectiveness in Egyptian children are lacking. To the best of our knowledge, this is the first study of its kind to be conducted in Egypt. Furthermore, there are gaps in current scientific knowledge regarding the management of SVT among the pediatric emergency nurses and the practical implementation of modified and standard Valsalva maneuvers. The performance of this maneuver, reduces dependency on antiarrhythmic drugs or electrical cardioversion for children with SVT and may also empower the parents to manage their children’s condition at home [18, 19]. Moreover, it could lessen the financial burden on families, and hospital management in developing countries with limited health care resources, such as Egypt. Therefore, this study will be beneficial in terms of economic efficiency.

*Aim of this study* was to compare the efficacy of modified versus standard Valsalva maneuvers on the clinical outcomes and satisfaction of children with paroxysmal supraventricular tachycardia.

## Methods

### Study design

This research employed a Randomized Controlled Trial Design (RCT).

### Participants

The study included children who met specific eligibility criteria. Eligible participants were cooperative children aged 7 to 17 years of both genders, who were hemodynamically stable and had a confirmed diagnosis of PSVT based on ECG. Participants were required to be willing to be in the study, and they should be free from communication difficulties or psychiatric disorders. The study excluded children with wide complex tachycardia, acute myocardial infarction, and severe pulmonary insufficiency, a history of aortic stenosis or glaucoma, heart failure, or inability to tolerate supine positioning or leg-raising movements.

### Setting

The study was conducted in the Pediatric Emergency Department and the Pediatric Cardiac Intensive Care Unit at Tanta University Hospitals, El-Gharbia Governorate, Egypt. *Pediatric Emergency Department*: This Department consists of a physician’s office and three patients’ rooms. The patient monitoring room contains five beds, each equipped with a monitor device and I.V. pole. It also contains a portable suction device and a crash cart equipped with a cardioversion device and emergency medications. The blood transfusion room contains fourteen automatic chairs with I.V. poles, and a hand-washing sink. The oxygen therapy and nebulizer session room contains seven beds, each with central suction and oxygen supply.

*Pediatric cardiac intensive care unit:* It consists of two sections, each containing two beds, a monitor device, and central suction and oxygen supply. It also contains two portable suction devices, a crash cart equipped with a cardioversion device, emergency medications, a hand-washing sink, a refrigerator, and two cupboards for essential equipment such as urinary catheters, nasogastric tubes, and sterile dressings.

**Fig. 2:**
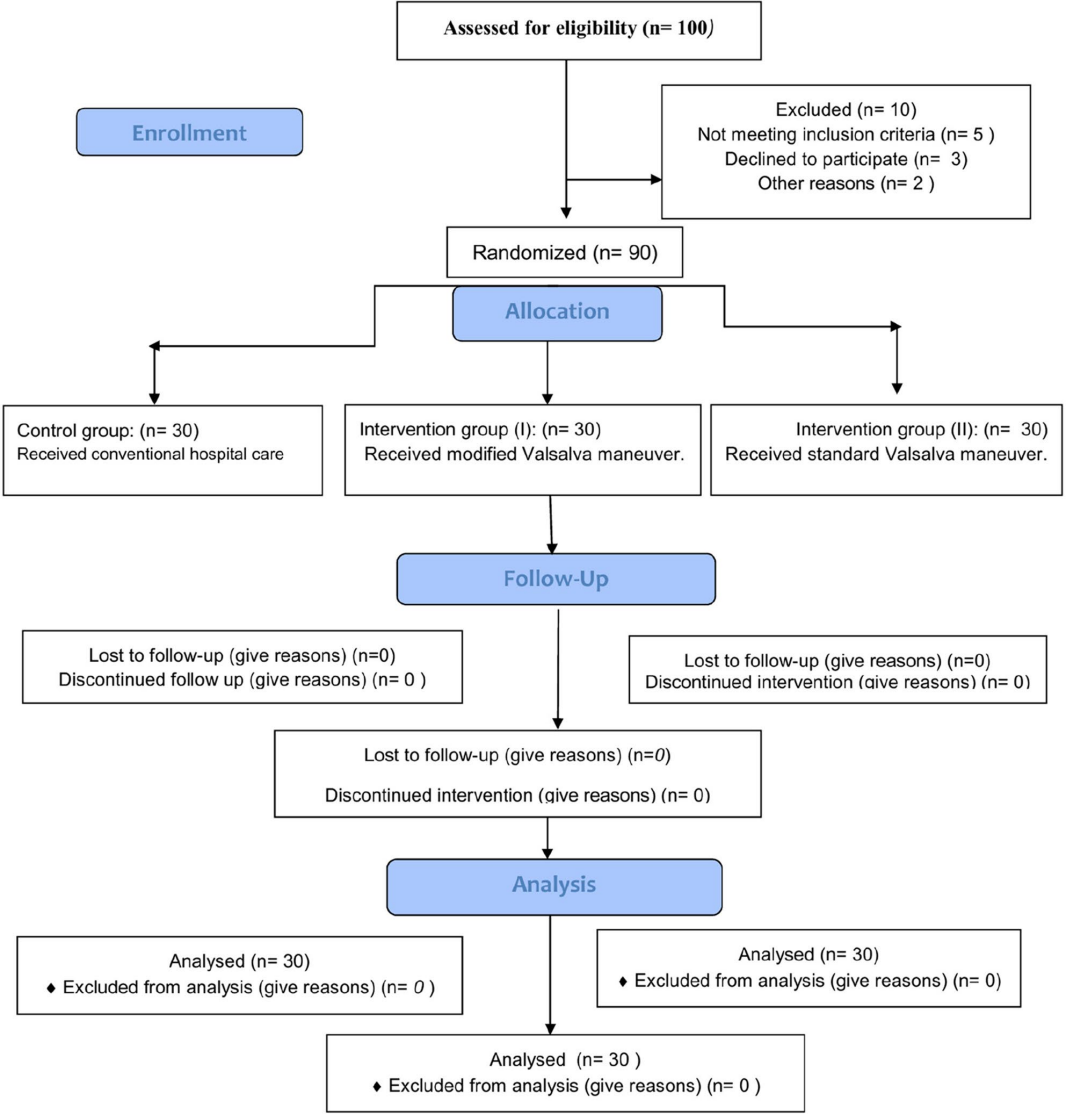
CONSORT flow diagram

*Data Collection*: From September 2024 to February 2025, the researchers visited the study settings four days a week (Saturday to Tuesday) from 10:00 am to 12:00 pm. The study design followed the CONSORT 2010 statement [20, 21] and included three groups (Fig. 2).

### Sample size determination

The researchers computed the sample size using G*Power software (version 3.1.9.7) [22] for a one-way ANOVA (fixed effects, omnibus) with α = 0.05. Assuming a medium effect size (Cohen’s f = 0.25), the achieved power is approximately 0.54, which indicates the study was underpowered to detect medium effects. For a large effect (Cohen’s f = 0.40) the achieved power would be ≈ 0.93. a t-test is used for independent samples with a power of 0.80 and a significance level of 0.05. The analysis indicated that at least of 80 children were needed for the study. The researchers selected equal allocation to maximize statistical power and simplify the analysis. The sample comprised three groups (*n* = 30 per group; total *N* = 90). The study employed a computer-generated randomization process using https://www.random.org/ to assign children to one of the three study groups: (Fig. 2 shows CONSORT flow diagram).

*-Control Group*: No intervention (Conventional Hospital Care)
*-The Intervention Group I*: (Modified Valsalva Maneuver plus Conventional Hospital Care)
*-The Intervention Group II*: (Standard Valsalva Maneuver plus Conventional Hospital Care)

### Ethical considerations

The Scientific Research Ethics Committee of the Faculty of Nursing, Tanta University, granted ethical approval for the study (Code No. 454-4−2024).

The researchers obtained informed consent from each participant’s parents or legal guardians, and reassured them that participation would not cause any harm or pain. The researchers informed participants of their right to participate or withdraw from the study at any time. The researchers maintained strict confidentiality and respected the privacy of the collected data.

#### Tools for data collection

**Fig. 3:**
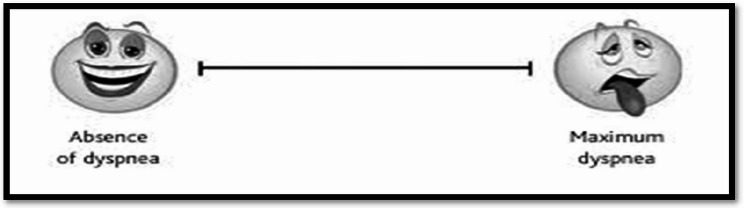
Visual Analogue Scale for dyspnea. Source: Crisafulli E, Clini EM. Measures of dyspnea in pulmonary rehabilitation. Multidiscip Respir Med. 2010; 5 (3):202–210. Doi: 10.1186/2049-6958-5-3-202

The researchers used four tools in the current study:

- *Tool I: A structured questionnaire*, divided into two parts collected the data:
- *Part 1*: Children’s socio-demographic data, including information such as gender, age, residence, educational level, and birth order.
- *Part 2*: Children' medical assessment data, which included anthropometric measurements (weight, height, and body mass index), current complaints at admission, and past medical history of coronary heart disease, diabetes, hypertension, valvular heart disease, and anemia.
- *Tool II: The children's follow-up form *includes physiological parameters such as heart rate, respiratory rate, blood pressure, and oxygen saturation levels.
- *Tool III: The visual analogue scale (VAS) for dyspnea*: The researchers used it to achieve a rapid classification of symptom severity such as pain and dyspnea. It is relatively easy to administer and well accepted by respondents, even in the critical care environment [23]. In this study, it was used to assess dyspnea on admission and after the implementation of the two maneuvers. It is a closed scale composed graphically of horizontal line that is 10 cm long and frequently had two ends, which were often indicated with dots, corresponding to two “pictures and/or verbal descriptors” that labelled at 0 with descriptor “absence of dyspnea” and at 100 with “maximum dyspnea” and there was no interval marker visible on the line (Fig. 3). Using VAS, The researchers instructed children to quantify their dyspnea by marking the point on the line that best corresponded to their symptom severity [24]. The researchers calculated VAS scores by measuring in centimeters from the start of the line to the center of the point the pediatric patient recorded. The researchers then interpreted theses scores as: absence of dyspnea (0), mild (1–3), moderate (4–6), severe (7–9) and maximum dyspnea (10), the reliability of the VAS was Cronbach α = 0.86.

- *Tool IV: The Short Assessment of Patient Satisfaction (SAPS) questionnaire*: The researchers used it to assess satisfaction of the studied children. It was designed by (Hawthorne et al. 2014) [25] and adopted by the researchers. It consisted of 7 items to evaluate the core domains of pediatric patients’ satisfaction, including maneuver satisfaction, explanation of maneuver results, clinician care, respect by the clinician, time with the clinician, and satisfaction with hospital care. Studies have shown that the SAPS is a valid and reliable measurement of patient satisfaction, as the reliability was Cronbach α = 0.86 [25]. The researchers categorized data using Likert scale five responses from 0 to 4 as the following, very dissatisfied (0), dissatisfied (1), neither satisfied nor dissatisfied (2), satisfied (3), very satisfied (4). The total score possible was 28. The researchers interpreted the scores as follows: 0 to 10 = very dissatisfied, 11 to 18 = dissatisfied, 19 to 26 = satisfied, 27 to 28 = very satisfied.

### A pilot study testing

The researchers conducted a pilot study with 10% of the sample size (nine children) who had PSVT in the above-mentioned settings to assess the applicability of the tools and the clarity of the related questions. The researchers introduced modifications, eliminating some questions, and adding others. The researchers didn’t include these children in the study sample.

*Primary Clinical Outcome: *The primary outcomes included SVT termination (return to sinus rhythm within the first 5 minutes of admission)

*Secondary Clinical Outcomes: *The secondary outcomes were the satisfaction of children, decreased dyspnea, reduced antiarrhythmic therapy use, and reduced length of stay time in hospital.

## Study procedures

### Data collection was carried out in three phases

#### Pre-intervention phase:

The researchers assessed all the children to ensure they met the inclusion criteria. For all three groups, researchers gathered and recorded children’s baseline socio-demographic and medical assessment data upon admission. Additionally, the primary outcomes were measured by the researchers using the children’s follow-up form and VAS for dyspnea on admission.

#### Intervention phase (Procedures)

The researcher positioned the children in the intervention group (I) who received the modified VM at an angle of 45° to 90° to the bed surface in a semi-recumbent position to facilitate normal breathing. Then, the researcher instructed the children to blow into 10 mL syringes and push the plunger until they reached the recommended intrathoracic pressure of 40 mmHg. They maintained the condition of exertion for 15 s after signs of acceptable movement appeared, such as jugular vein filling, increased abdominal muscle tension, and flushing. The researcher then abruptly placed the child in the supine position with his or her legs elevated at a 45° angle for 15 s. The children then returned to the semi-recumbent position and remained there for another 45 s. They were reevaluated for cardiac rhythm return initially through ECG monitoring to determine cardioversion [10, 26].

 Children who received the standard VM in an intervention group (II) were positioned in a semi-recumbent position. The angle was between 45° and 90° relative to the bed surface. The researcher asked the child to take a deep breath, seal their lips tightly around a 10 ml syringe, and attempt to move the plunger by blowing into the syringe for 15 s. Then, the researcher told the children to relax and resume normal breathing, and to remain in the same position for 60 s before the researcher reassessed their cardiac rhythm, initially by ECG monitoring to determine the cardioversion [27].

Children in the control group received conventional hospital care and all data collection steps were applied in the same manner as in the intervention groups.

#### Post intervention phase (Outcomes Measurements)

The researchers reassessed the outcomes using the children’s follow-up form and VAS for dyspnea at 1, 3, and 5 min post the implementation of the two maneuvers. Each intervention was performed for a maximum of 3 attempts at one minute intervals before switching to antiarrhythmic medication according to the guidelines, except the return of sinus rhythm was recorded by the treating physician and confirmed by ECG [28]. The children’s satisfaction with the two maneuvers was measured using the SAPS, after sinus rhythm restoring at 5 min after the implementation of the two maneuvers.

### Statistical analysis

The researchers used the statistical Package for the Social Sciences (SPSS), version 26, (SPSS Inc., Chicago, IL, USA), to organize, and analyze the collected data statistically. For quantitative data, the researchers calculated the range, mean, and standard deviation.

For qualitative data, which describe a categorical set of data by frequency, percentage, or proportion of each category, the researchers performed a comparison between two groups and more using Chi square test (χ2). Regarding, comparison between more than two means of parametric data, the researchers calculated F value of ANOVA test and the comparison between means of three or more related groups (on admission, at one, three, and five minutes) χ2 value of Friedman test for non-parametric data. For the interpretation of the results of the significance tests, a P-value less than 0.05 was considered significant and a P-value less than 0.001 was considered highly significant [29].

## Results

Table 1 shows that mean age of the children in the control, MVM, and SVM groups were 13.43 ± 2.76, 13.50 ± 2.82, and 13.60 ± 2.51 years, respectively. Nearly two-thirds of the children were female. The three groups were also homogenous in terms of socio-demographic characteristics, showing no statistically significant difference between them. Regarding the number of Valsalva maneuver attempts, about 56.3% of children who received MVM and 40% of children received SVM had one attempt. This is followed by 37.5% and 30% of children in MVM and SVM groups respectively had two attempts. In addition, the least percentage 6.2% of MVM group versus 30% of SVM group displayed three attempts.

**Table 1:** The studied children’s socio-demographic data and numbers of Valsalva maneuver attempts (*n* = 90)

Variables	Control group (*n* = 30)			Modified Valsalva maneuver Group (I) (*n* = 30)			Standard Valsalva maneuver Group (II) (*n* = 30)		χ 2 *P*
	No	%		No	%		No	%	
Age (years):									
7–12 > 12–17 Mean ± SD	8 22	26.7 73.3		9 21	30.0 70.0		7 23	23.3 76.6	0.341 0.843
	**13.43 ± 2.76**			**13.50 ± 2.82**			**13.60 ± 2.51**		**F value**,** P** 0.029, 0.972
Gender									
Male	13		43.3	12		40.0	11	36.7	0.270
Female	17		56.7	18		60.0	19	63.3	0.870
Residence:									
Rural	20		66.7	17		56.7	22	73.3	1.870
Urban	10		33.3	13		43.3	8	26.7	0.393
Educational level:									
Primary school	8		26.7	7		23.3	4	13.3	
Preparatory school	12		40.0	13		43.4	15	50.0	1.783
Secondary school	10		33.3	10		33.3	11	36.7	0.776
Birth order									
First	17		56.7	14		46.7	15	50.0	
Second	11		36.6	15		50.0	13	43.3	1.320
Third	2		6.7	1		3.3	2	6.7	0.858
No. of attempts needed for success:				**No. = 16**		**%**	**No. = 10**	**%**	
1	0		0.0	9		56.3	4	40.0	**Group (I) Vs (II)** 2.681
2	0		0.0	6		37.5	3	30.0	
3	0		0.0	1		6.2	3	30.0	0.262

# More than one symptom * Significant Difference at (*P*˂0.05)

Table 2 shows that palpitations were the most prevalent symptom among children who had active complaints on admission 63.3%, 70%, and 60% of participants in the control, MVM, and SVM groups, respectively. The next most common symptom was dyspnea, affecting 26.7%, 16.7%, and 33.3% of children in the control, MVM, and SVM groups, respectively. Additionally, more than two-thirds of the children in each group had previous episodes of PSVT: 73.3% in the control group, 66.7% in the MVM group, and 63.3% in the SVM group. Also, it was evident that all children in the control group (100%) received antiarrhythmic drugs for management of the episodes, compared with 46.7% and 66.7% of the children within the MVM and SVM groups, respectively. Moreover, statistically significant differences were observed among the three groups regarding length of hospital stay (*p* = 0.002). Length of hospital stay differed significantly across groups (F = 6.614, *P* = 0.002). Children in the modified maneuver group had the shortest average hospital stay (1.23 ± 0.43 h), significantly less than both the control (1.83 ± 0.74 h) and standard maneuver groups (1.73 ± 0.82 h).

**Table 2:** The studied children’s medical assessment data (*n* = 90)

Children’s medical assessment data		Control group (*n* = 30)			Modified Valsalva maneuver Group (I) (*n* = 30)		Standard Valsalva maneuver Group (II) (*n* = 30)		χ 2 *P*
		No	%		No	%	No	%	
Past medical history of: #									
Congenital heart lesions		2	6.7		2	6.6	1	3.3	
Anemia		10	33.3		12	40.0	13	43.3	
Diabetes mellitus		5	16.7		6	20.0	3	10.0	
Hypertension		4	13.3		2	6.7	3	10.0	3.752
Valvular heart disease		1	3.3		2	6.7	3	10.0	0.958
Gastrointestinal disease		8	26.7		6	20.0	7	23.4	
Children’s complaints at admission #									
Palpitation	19			63.3	21	70.0	18	60.0	
Dyspnea	8			26.7	5	16.7	10	33.3	4.593
Chest pain	4			13.3	8	26.7	5	16.7	0.800
Pallor	6			20.0	5	16.7	9	30.0	
Sweating	3			10.0	4	13.3	5	16.7	
Previous episodes of Paroxysmal supraventricular tachycardia (PSVT)									
Yes	22			73.3	20	66.7	19	63.3	0.712
No	8			26.7	10	33.3	11	36.7	0.700
Administered antiarrhythmic drugs									
Yes	30			100.0	14	46.7	20	66.7	**21.202**
No	0			0.0.	16	53.3	10	33.3	0.001**
Length of stay time in hospital (Hours)									
1	11			36.7	23	76.7	15	50.5	
2	13			43.3	7	23.3	8	26.7	**13.401**
3	6			20.0	0	0.0	7	23.3	0.009*
Mean ± SD	**1.83 ± 0.74**				**1.23 ± 0.43**		**1.73 ± 0.82**		**F value**,** P** **6.614**, 0.002*

# More than one answer

*Significant Difference at (*P*˂0.05), ** Highly Significant Difference at (*P*˂0.001)

Table 3 illustrates that, among the three groups, children in the MVM group had the lowest mean scores for blood pressure, heart rate, and respiratory rate at 1, 3, and 5 min post-implementation of the maneuver. The heart rate showed a marked decline in the intervention groups, particularly in the modified maneuver group, where the effect size reached a large magnitude at five minutes (d = 0.841). Higher mean oxygen level values were observed at 95.03 ± 0.49, 97.40 ± 1.00, and 99.10 ± 0.54 in the MVM group at one, three, and five minutes post-implementation of maneuvers, respectively. This indicates an elevated oxygen level in the MV group compared to the other two groups. There is a highly statistically significant difference between the three groups (*P* = 0.0001).

**Table 3:** Mean scores of the studied children’s physiological parameters (*n* = 90)

Children’s Physiological parameters	Control group (*n* = 30)	Modified Valsalva maneuver Group (I) (*n* = 30)	Standard Valsalva maneuver Group (II) (*n* = 30)	F value *P*		Effect size	
	Range Mean ± SD	Range Mean ± SD	Range Mean ± SD			Cohen’s d	level
Systolic blood pressure (mm Hg)							
On admission	146.83 **±** 11.17	147.50 **±** 11.42	146.83 **±** 8.75		0.040 0.961	0.001	Very small
At one minute	141.00 **±** 7.11	135.00 **±** 6.82	140.50 **±** 5.62		**7.740** 0.001**	**0.151**	**Small**
At-three minute	132.00 **±** 6.51	124.33 **±** 4.30	129.66 **±** 4.90		**16.364** 0.001**	**0.273**	**Medium**
At-five minute	126.33 **±** 4.72	118.66 **±** 3.45	121.16 **±** 5.82		**20.177** 0.001**	**0.317**	**Medium**
**χ 2 value**,** P**	**72.801**, 0.001**	**82.702**, 0.001**	**79.785**, 0.001**				
Diastolic blood pressure (mm Hg)							
On admission	103.00 **±** 8.36	101.66 **±** 9.85	101.33 **±** 8.19		0.299 0.742	0.007	Very small
At one minute	97.66 **±** 5.83	87.00 **±** 7.02	91.66 **±** 7.46		**18.502** 0.001**	**0.298**	**Medium**
At-three minute	95.66 **±** 5.04	81.33 **±** 4.34	85.50 **±** 5.92		**61.666** 0.001**	**0.586**	**Medium**
At-five minute	91.83 **±** 3.82	79.33 **±** 8.27	80.83 **±** 7.66		**29.546** 0.001**	**0.404**	**Medium**
**χ 2 value**,** P**	**62.884**, 0.001**	**60.316**, 0.001**	**75.435**, 0.001**				
Heart rate (Beats/min)							
On admission	176.00 **±** 15.53	175.10 **±** 16.09	173.53 **±** 14.92		0.194 0.824	0.004	Very small
At one minute	167.16 **±** 15.17	129.03 **±** 23.29	140.66 **±** 20.59		**27.827** 0.001**	**0.375**	**Medium**
At-three minute	156.40 **±** 14.73	103.83 **±** 6.23	117.13 **±** 16.00		**131.231** 0.001**	**0.744**	**Medium**
At-five minute	143.13 **±** 13.99	93.83 **±** 4.89	103.06 **±** 12.44		**165.018** 0.001**	**0.841**	**Large**
**χ 2 value**,** P**	**88.349**, 0.001**	**90.000**, 0.001**	**89.101**, 0.001**				
Respiratory rate (Breath/min)							
On admission	37.06 **±** 1.83	36.36 **±** 1.62	36.83 **±** 2.08		1.102 0.337	0.025	Very small
At one minute	33.03 **±** 2.15	**28.80 ± 2.09**	30.76 **±** 3.22		**20.800** 0.001**	**0.323**	**Medium**
At-three minute	31.76 **±** 1.97	**24.56 ± 1.07**	26.63 **±** 2.77		**97.027** 0.001**	**0.690**	**Medium**
At-five minute	29.73 **±** 1.63	**22.36 ± 1.96**	23.63 **±** 2.04		**136.290** 0.001**	**0.758**	**Medium**
**χ 2 value**,** P**	**87.932**, 0.001**	**87.976**, 0.001**	**88.946**, 0.001**				
Oxygen levels (%)							
On admission	92.90 **±** 0.99	92.60 **±** 1.24	92.96 **±** 1.03		0.949 0.391	0.021	Very small
At one minute	94.33 **±** 0.99	**95.03 ± 0.49**	94.66 **±** 0.58		**7.223** 0.001**	**0.142**	**Medium**
At-three minute	94.46 **±** 0.57	**97.40 ± 1.00**	96.50 **±** 0.97		**89.075** 0.001**	**0.672**	**Medium**
At-five minute	95.40 **±** 0.72	**99.10 ± 0.54**	97.93 **±** 0.78		**222.631** 0.001**	**0.837**	**Large**
**χ 2 value**,** P**	**69.688**, 0.0001**	**87.962**, 0.0001**	**87.031**, 0.0001**				

* Significant Difference at (*P*˂0.05), ** Highly Significant Difference at (*P*˂0.001). χ 2 value of Friedman test

**Fig. 4:**
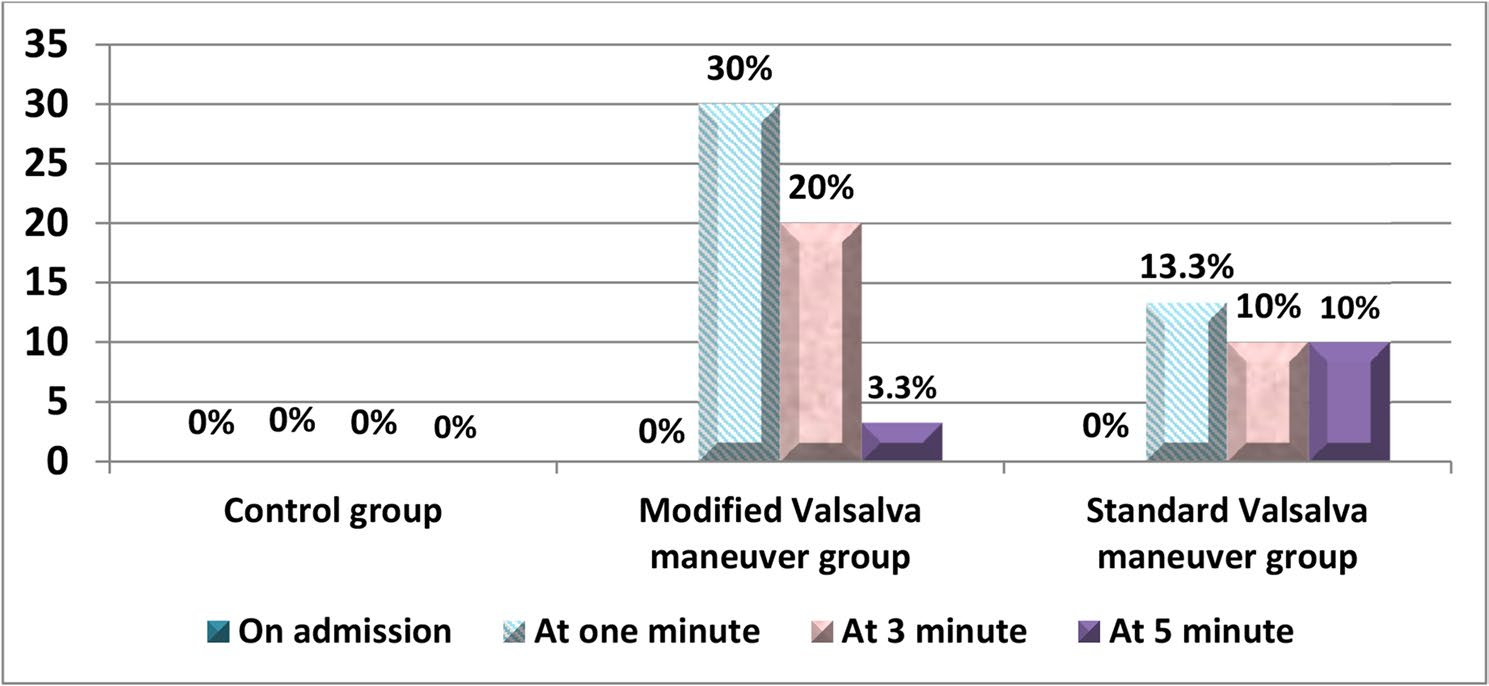
Percentage distributions of studied children’s sinus rhythm return

Fig. 4: The control group showed no successful conversions at any point (0%). In comparison, the MVM group achieved the highest success rates, with 30% conversion at one minute, 20% at three minutes, and 3.3% at five minutes, indicating a quick and effective outcome. Meanwhile, the SVM group demonstrated lower conversion rates, 13.3% at one minute and 10% at both three and five minutes, suggesting a moderate but slower improvement compared to the modified maneuver. Overall, more than half 53.3%, of the children in MVM group compared to 33.3% of the children who received the SVM group returned to normal sinus rhythm within the first five minutes post-implementation.

Table 4 shows the percentage distribution of children’s dyspnea and satisfaction levels across the three groups. On admission, the majority of participants (83.3%, 76.7%, and 86.7% in the control, MVM, and SVM groups, respectively) experienced severe dyspnea. One minute after the maneuvers were performed, the severity of dyspnea level decreased from severe to moderate in 36.7%, 90%, and 93.3% of children in the control, MVM, and SVM groups, respectively. By the third minute, mild dyspnea was observed in 3.3%, 76.7%, and 93.3% of children in the respective groups. At the fifth minute, nearly all children in the MVM group (96.7%) reported no dyspnea, compared to 33.3% in the SVM group and none in the control group, reflecting a moderate effect size. Additionally, satisfaction levels were significantly higher in the modified maneuver group, indicating greater perceived effectiveness. Highly significant differences in mean dyspnea scores were observed among the three groups at 1, 3, and 5 min post intervention.

**Table 4:** Percentage distribution of children’s dyspnea and satisfaction levels among the three groups toward the maneuvers (*n* = 90)

A) Visual analogue scale for dyspnea							χ 2 *P* (I)	χ 2 *P* (II)	χ 2 *P* (III)	Effect size Cohen’s d, level
	Control group (*n* = 30)		Modified Valsalva maneuver Group (I) (*n* = 30)		Standard Valsalva maneuver Group (II) (*n* = 30)					
	No.	%	No.	%	No.	%				
On admission										
Moderate dyspnea	3	10.0	4	13.3	3	10.0				
Severe dyspnea	25	83.3	23	76.7	26	86.7	0.426	0.353	1.327	0.024
Maximum dyspnea	2	6.7	3	10.0	1	3.3	0.808	0.838	0.515	Very small
At one minute										
Mild dyspnea	0	0.0	3	10.0	2	6.7	**28.73**	**28.41**	0.218	0.415
Moderate dyspnea	11	36.7	27	90.0	28	93.3	**0.001**	**0.001**	0.640	Medium
Severe dyspnea	19	63.3	0	0.0	0	0.0	**	**		
At-three minute										
Absence dyspnea	0	0.0	5	16.6	0	0.0				
Mild dyspnea	1	3.3	23	76.7	28	93.3	**49.00**	**48.98**	5.49	0.390
Moderate dyspnea	17	56.7	2	6.7	2	6.7	**0.001**	**0.001**	0.064	Medium
Severe dyspnea	12	40.0	0	0.0	0	0.0	**	**		
At-five minute										
Absence dyspnea	0	0.0	29	96.7	10	33.3	**56.33**	**31.61**	**26.44**	**0.727**
Mild dyspnea	11	36.7	1	3.3	20	66.7	**0.001**	**0.001**	**0.001**	Medium
Moderate dyspnea	19	63.3	0	0.0	0	0.0	**	**	**	
B) Satisfaction levels										
Very dissatisfied	-	-	0	0.0	0	0.0			**18.095**	**0.301**
Dissatisfied.	-	-	0	0.0	8	26.7			**0.0001**	**Medium**
Satisfied.	-	-	20	66.7	22	73.3			**	
Very satisfied	-	-	10	33.3	0	0.0				

(I) Control v/s Modified (II) Control v/s standard (III) Modified v/s standard

* Significant Difference at (*P*˂0.05) ** Highly Significant Difference at (*P*˂0.001)

Table 5 presents that on admission, there were no significant differences in dyspnea mean scores among the three groups (*p* = 0.758). However, at one, three, and five minutes following the intervention, both Valsalva maneuver groups showed a significant reduction in dyspnea scores compared to the control group (*p* < 0.001). The effect sizes were within the medium range, indicating a meaningful improvement, particularly in the MVM. Additionally, the satisfaction scores was significantly higher in the MVM group (Mean = 25.56 ± 1.67) compared with the SVM group (Mean = 20.10 ± 2.57; t = 9.740, *p* < 0.0001).This shows that MVM produced a mean satisfaction score of 25.56 ± 1.67 that was higher than the SV maneuver’s score of 20.10 ± 2.57, with highly statistically significant differences (*P* = 0.0001).

**Table 5:** Comparison between children’s mean scores of dyspnea and satisfaction toward the maneuvers for the three groups (*n* = 90)

Variables	Control (*n* = 30)	Modified Valsalva maneuver (*n* = 30)	Standard Valsalva maneuver (*n* = 30)		Effect size	
Total score of dyspnea	Mean ± SD	Mean ± SD	Mean ± SD	F value, *P*	Cohen’s d	level
On admission	7.80 **±** 1.12	7.70 **±** 1.34	7.56 **±** 1.16	0.278, 0.758	0.006	Very small
At one minute	6.80 **±** 1.15	4.70 ± 1.02	4.96 ± 0.86	37.876,0.001**	0.465	Medium
At-three minute	6.00 **±** 1.17	2.16 ± 1.20	2.83 ± 0.53	121.19, 0.0001**	0.736	Medium
At-five minute	3.96 **±** 1.29	0.03 ± 0.18	1.56 ± 1.25	107.642, 0.001**	0.712	Medium
B) Satisfaction score						
**Mean ± SD**	---	25.56 ± 1.67	20.10 ± 2.57	**t-test** **9.740**, 0.0001**	0.621	Medium

* Significant Difference at (*P*˂0.05), ** Highly Significant Difference at (*P*˂0.001)

## Discussion

The management of (PSVT) in children presents a clinical challenge due to its abrupt start and termination. Therefore, there is an urgent need for practical, safe methods to convert PSVT to normal sinus rhythm in pediatric emergency situations. The present study results revealed that palpitation was the most frequent complaint reported by children at the time of admission. Yılmaz et al. (2022) agreed with this result, as they reported that palpitation was the most common reason and complaint, at 59.4%, in patients diagnosed with SVT in the pediatric cardiology clinic [30]. Also, this was in congruency with another study by Rotés et al. (2020) which aimed to establish the incidence and describe the clinical presentation, prognosis and treatment of (SVT) as a main reason for between-hospital transfer in children. They found that 81.2% of the children in the study had palpitation, which was the symptom that led to the diagnosis [31].

Antiarrhythmic drugs are commonly used to manage PSVT; however, there are risks associated with their use, including the potential for decreased blood pressure, serious heart rhythm disturbances, and even cardiac arrest [6]. Interestingly, in this study the application of Valsalva maneuvers reduced the necessity of antiarrhythmic drugs use, and their undesirable effects, in about 53.3% and 33.3% of the children in the MVM and SVM groups, respectively. On the other hand, 46.7% and 66.7% of the children in the MVM and SVM groups, respectively, still required antiarrhythmic drugs; this might be due to the fact that after three unsuccessful attempts, clinicians should move on to other pharmacological interventions, such as intravenous adenosine. In a case study Smith et al., (2017) [32] supported these results and described the MVM as being superior to IV cannulation and antiarrhythmic therapy in children. Moreover, children in the MVM group had significantly shorter stay duration in the hospital compared to children in the SVM and control groups. This finding aligned with previous studies by Lan et al., (2021) and Ferreira et al., (2021) who concluded in their systematic review that MVM reduced the use of antiarrhythmic drugs and did not increase time spent in Emergency Department [19, 26]. In contrast, two studies by Ashraf et al.(2023) and Chen et al. (2020) found no statistically significant difference between the standard and modified Valsalva maneuvers in terms of the duration of hospital stay [14, 33].

Regarding physiological parameters of the studied children, the MVM group had lower mean scores of physiological indices than the other two groups at the 1, 3, and 5 min post-implementation of the maneuvers, with highly statistically significant differences. These differences indicated the efficiency of MVM in decreasing the rate of these physiological indices. From the researchers’ perspective, passively elevating the legs in the MVM maneuver increases blood return to the heart, which subsequently raises jugular vein pressure rises. The rise in pressure has three effects: it increases vagal tone, stimulates the vagus nerve, and decreases heart rate [34]. This result was congruent with a previous study carried out in Pakistan, which found that the MVM group had lower mean scores for systolic and diastolic blood pressure, as well as heart rate, at the beginning and end of the intervention compared with the SVM group [14]. Similarly, research by Balgote & Deshkar (2019) indicated that after practicing modified Valsalva maneuver, their participants’ heart rate reduced significantly [35]. This result was in contrast to a study result, which reported that there were no differences among the groups regarding vital signs parameters [36].

**Fig. 5:**
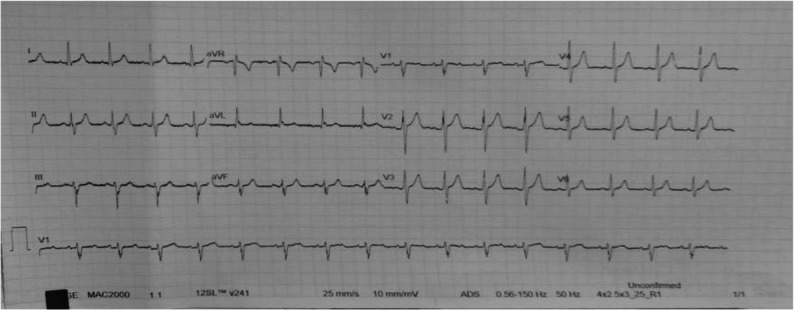
a 12 leads ECG shows normal sinus rhythm after MVM implementation for a real case in this study

In addition, the findings of the present study indicated that more than half of the children who received MVM (53.3%) returned to sinus rhythm compared with the 33.3% of children who received SVM within the first five minutes post-implementation as documented by 12 leads ECG and shown in (Fig. 5), which indicated that the success rate of the MVM group was higher than the SVM group. The rationale could be related to that the modified Valsalva technique can boost left atrial pressure and activate the carotid baroreceptors synergistically to end supraventricular tachycardia and restore sinus rhythm by rapidly increasing the pressure in the thoracic cavity and the volume of returned heart blood [37].

The current results were approved by Uysal et al. (2023) who concluded that 30% of their studied children were treated with vagal maneuvers and 61% of them had their SVT attacks terminated [38]. In the same line Lewis et al., (2017) found that vagal maneuver was successful in acute episodes management in 25% of pediatric supraventricular tachycardia [39]. Similar study supported the current result and reported that supraventricular tachycardia was terminated with vagal maneuver in 23% of pediatric patients [30]. According to another prior study that assessed the cardiac rhythm following the application of Valsalva maneuvers, 20% of participants in the SVM group returned to sinus rhythm at one min, compared with 58% of participants in the MVM group [14]. Additionally, the current results were approved by Lan et al. (2021) who stated that success rate of achieving sinus rhythm in patients with MVM was higher than that in patients with SVM [26]. From the researchers’ point of view, the factors that might influence success rate could be age, technique adherence, and numbers of maneuver attempts.

Regarding dyspnea, our results revealed that the majority of children in the three groups suffering from severe dyspnea on admission, and post-intervention, the degree of dyspnea significantly decreased from severe to moderate in most children within the MVM, and SVM groups. Notably, the severity of dyspnea decreased until it reached mild dyspnea at 3 min post- intervention. This reduction may occur because the modified Valsalva generates a constant decline in blood pressure as a limited volume of blood returns to the heart, thereby decreasing heart’s workload and consequently reducing dyspnea. From the researchers’ perspective, performing the MVM can improve dyspnea in SVT patients by helping to convert the SVT to sinus rhythm and consequently reducing symptoms. The findings of this study were confirmed by the results of a study [40] that concluded that a significant improvement and decrease in the severity of dyspnea among study group after implementing of MVM.

The current study revealed that children who received MVM have a higher mean satisfaction score than those who received SVM, with highly statistically significant differences. This finding may be explained on the basis of, the modified Valsalva technique is simple to apply, noninvasive, easy to use, and sufficiently safe [10]. Moreover, it was less distressing for children, because there was no need for intravenous cannulation for administrating IV adenosine. A previous study [32], supported the current result, reporting that posture modification during Valsalva technique, when applied within the first five minutes of arrival, successfully restored the sinus rhythm to normal on the first attempt, leading to improved children satisfaction score. Additionally, the current results were supported by Suárez et al. (2024), who stated that the modified Valsalva maneuver allows for non-invasive management of supraventricular tachycardia without pharmacological intervention or electrical cardioversion in pediatric cardiac surgeries [41]. In contrast, Wang et al. (2020) found that the rates of acceptance among participants in the modified VM group and the standard VM group were not significantly different [37].

### Limitations

One of the study’s limitations was the small sample size, and data were collected from a single center, which could affect the generalizability of the results. In addition, the study used non-blinded design that could introduce some bias. The study setting declined permission for any photographs to be taken during the procedure, and limited photography to ECG recordings only.

### Conclusion

Comparing the efficacy of the two maneuvers on clinical outcomes in children with paroxysmal supraventricular tachycardia (PSVT) revealed that the modified Valsalva maneuver was significantly more effective than the standard version in terminating PSVT. This effectiveness led to better children’s clinical outcomes in terms of decreasing the degree of dyspnea within the first minute from severe to moderate and reducing the need for administering antiarrhythmic drugs for management of SVT episodes. Additionally, there were statistically significant differences in hospital stay duration, with children in the MVM group had notably shorter stays duration than those in the SVM group. Moreover, children in the MVM group had a higher mean satisfaction score than those in SVM group, with highly statistically significant differences.

### Recommendations

The Modified Valsalva maneuver is simple and easy to operate for children. Therefore, it can be performed as first-line therapy for children with stable PSVT at Emergency Departments prior to the use of pharmacological interventions. It is recommended to train nursing staff, children’s parents, and older children on how to apply the maneuver at home. Further research is needed, for investigating the potential adverse side effects with such maneuvers in children and multi-center studies are also recommended in the future.

### Implications for practice

This study points out important considerations for pediatric nurses, especially in the management of hemodynamically stable paroxysmal supraventricular tachycardia (PSVT) in children. The Modified Valsalva maneuver (MVM) demonstrates a greater effectiveness than the standard Valsalva maneuver in restoring normal sinus rhythm without the use of medications. This finding emphasizes the need for educating and training emergency nurses on the proper maneuver and the underlying physiology of MVM. Implementing this safe, non-invasive, and cost-effective maneuver as an initial treatment can lead to better children’s outcomes, decrease dependence on antiarrhythmic drugs or electrical cardioversion, and minimize the risk of associated complications. In addition, integrating MVM training into Emergency Department guidelines may enhance the quality and safety of SVT management in children and increase their satisfaction with the care.

### Data Availability

The data that support the findings of this study are available. They are available upon request from the corresponding author. These data are not publicly available due to privacy or ethical restrictions.

## Supplementary Information

Supplementary Material 1.

Supplementary Material 2.

Supplementary Material 3.

Supplementary Material 4.

## Data Availability

The data that support the findings of this study are available. They are available upon request from the corresponding author. These data are not publicly available due to privacy or ethical restrictions.

## Abbreviations

PSVT: Paroxysmal supraventricular tachycardia
ECG: Electrocardiography
MVM: Modified valsalva maneuver
SVM: Standard valsalva maneuver
AVNRT: Atrioventricular nodal reentrant tachycardia
VAS: Visual analogue scale
