# Supplementary material for: Efficacy of modified versus standard Valsalva maneuvers on clinical outcomes and satisfaction of children with paroxysmal supraventricular tachycardia: randomized control trial
Source: BMC Pediatr. 2025 Dec 17;25:1003. doi: 10.1186/s12887-025-06396-9 (PMC12752250; doi:10.1186/s12887-025-06396-9)
Supplement: Supplementary file 1 — Supplementary Material 1. [file 12887_2025_6396_MOESM1_ESM.doc]

**CONSORT 2010 Flow Diagram**

**Allocation**

**Analysis**

**Follow-Up**

**Enrollment**

Assessed for eligibility (n= 179)

Excluded (n= 19 )

♦  Not meeting inclusion criteria (n= 7 )

♦  Declined to participate (n= 12)

Lost to follow-up (give reasons) (n= 0 )

Discontinued follow up (give reasons) (n= 0 )

Group (2) Virtual reality (n=40)

Lost to follow-up (give reasons) (n= 0 )

Discontinued intervention (give reasons) (n= 0)

Group (3) Buzzy Bee (n=40)

Randomized (n= 90)

Group (1) Control

(n=40)

Lost to follow-up (give reasons) (n= 0 )

Discontinued intervention (give reasons) (n= 0)

Analysis (n=160)

Excluded from analysis (n=0)

Group (4) Kaliodescope (n=40)
