# Supplementary material for: Efficacy of modified versus standard Valsalva maneuvers on clinical outcomes and satisfaction of children with paroxysmal supraventricular tachycardia: randomized control trial
Source: BMC Pediatr. 2025 Dec 17;25:1003. doi: 10.1186/s12887-025-06396-9 (PMC12752250; doi:10.1186/s12887-025-06396-9)
Supplement: Supplementary file 3 — Supplementary Material 3. [file 12887_2025_6396_MOESM3_ESM.docx]

**Supplementary result**

**Figure (1): Mean score of studied children related to Visual analogue scale (VAS) for dyspnea (n=90)**

**Tabl 1 Percentage distribution and mean score of studied children of modified and standard Valsalva maneuver related to their satisfaction (n=60)**

| **Satisfaction** | **The studied children (n=90)** | | | | **χ 2**  **P** |
| --- | --- | --- | --- | --- | --- |
|  | **Modified Valsalva maneuver**  **Group (I) (n=30)** | | **Standard Valsalva maneuver**  **Group (II) (n=30)** | |  |
|  | **No.** | **%** | **No.** | **%** |  |
| **Satisfaction levels** |  | | | |  |
| Very dissatisfied | 0 | 0.0 | 0 | 0.0 |  |
| Dissatisfied. | 0 | 0.0 | 8 | 26.7 | **18.095** |
| Satisfied. | 20 | 66.7 | 22 | 73.3 | **0.0001**** |
| Very satisfied | 10 | 33.3 | 0 | 0.0 |  |

**** Highly Significant Difference at (P˂0.001),**

**Figure (2): Mean score of studied children of modified and standard Valsalva maneuver related to their satisfaction (n=60)**

**Table 2 Relationship between age, gender and type of maneuvers**

| **Variables** | | **Total (n = 60)** | **Modified Valsalva maneuver**  **Group (I)**  **(n=30)** | **Standard Valsalva maneuver**  **Group (II) (n=30)** | **P value** |
| --- | --- | --- | --- | --- | --- |
|  |  | **Mean ± SD** | | |  |
| **Age** | | 13.55 ± 0.070 | 13.50 ± 2.82 | 13.60 ± 2.51 | 0.941 |
| **Gender** | **Male** | n=23 (38.3%) | n=12 (40.0%) | n=11 (36.7%) | 0.791 |
|  | **Female** | n=37 (61.7%) | n=18 (60.0%) | n=19 (63.3%) |  |
